# Evaluation of Hippo Pathway and CD133 in Radiation Resistance in Small-Cell Lung Cancer

**DOI:** 10.1155/2021/8842554

**Published:** 2021-01-13

**Authors:** Kui Yang, Yang Zhao, Yonghao Du, Ruixiang Tang

**Affiliations:** ^1^Department of General Surgery, The First Affiliated Hospital of Xi'an Jiaotong University, Xi'an 710000, China; ^2^Department of Oncology, Southwest Hospital, Third Military Medical University, Chongqing 400000, China; ^3^Department of Radiology, The First Affiliated Hospital of Xi'an Jiaotong University, Xi'an 710000, China; ^4^Department of Oncology Surgery, The First Affiliated Hospital of Xi'an Jiaotong University, Xi'an 710000, China

## Abstract

Although the Hippo pathway and CD133 have been reported to play pertinent roles in a variety of cancer, knowledge about their contribution to radiation resistance in small-cell lung cancer (SCLC) is limited. In this first-of-a-kind study, we have reported the expression of key Hippo pathway proteins in SCLC patients by immunohistochemical staining. We assessed the involvement of yes-associated protein 1 (YAP1) in radiation resistance by Cell Counting Kit-8 (CCK-8) and flow cytometry. In addition, we analysed the impact of CD133 on radiotherapy for SCLC. The mammalian Ste20-like serine/threonine kinase 2(MST2), pMST2, and pYAP1 in the Hippo pathway were not significantly associated with the disease stage and survival time in patients with SCLC. However, the pYAP1 expression showed some significance in the “YAP/TAZ subgroup” of SCLC patients. The proportion of CD133 in the SCLC cells was controlled by the YAP1 expression. The CD133 and YAP1 levels were significantly correlation with each other in tissues of SCLC patients. We sorted and isolated the CD133^+^ and CD133^−^cells in H69 and found that the cell surface glycoprotein may be associated with the radiation resistance of SCLC.In summary, we have firstly reported the expression of key Hippo pathway proteins in SCLC patients. Furthermore, we also identified that CD133 may be controlled by the expression of YAP1 in the Hippo pathway and that CD133 may be associated with the radiation resistance of SCLC.

## 1. Introduction

Small-cell lung cancer (SCLC) is an aggressive lung cancer subtype with high recurrence rates and poor outcomes [1]. Despite the progressions made in immunotherapy, the major treatment modality for SCLC remains to chemotherapy and radiotherapy, regardless of the status being limited disease (LD) or extensive disease (ED) [2, 3]. However, most of the SCLC patients ultimately develop chemotherapy or/and radiation resistance [4]. Hence, it is important to understand the underlying mechanisms for this resistance to achieve a breakthrough in treatment.

The Hippo signaling cascade plays various roles in cancer, including the regulation of cell proliferation and apoptosis, progression regulation of stem cell self-renewal, drug resistance, and metastasis [5–7]. The mammalian Ste20-like serine/threonine kinase1/2 (MST1/2) in this cascade can be phosphorylated by external stimuli such as KIBRA/NF2 and TAO kinases [8, 9]. Large tumor-suppressor kinase 1/2 (LATS1/2) on the downstream can be phosphorylated by MST1/2. Then, LATS1/2 phosphorylates the core proteins yes-associated protein (YAP)/transcriptional coactivator with PDZ-binding motif (TAZ) [10]. The YAP/TAZ functionality is controlled via localization within the cells as well as phosphorylation. While the phosphorylated YAP/TAZ are restricted and degraded in the cytosol, their dephosphorylation allows them to enter the nucleus and interact with the key transcription factors, thus driving tumor proliferation and metastasis [11]. The Hippo pathway is important in various forms of cancers, including in non-SCLC (NSCLC), breast, colon, liver, and stomach cancers [12]. However, the information about this pathway with regard to SCLC is limited.

CD133 is a well-known marker of cancer stem cells (CSCs) in several malignancies such as NSCLC [13], ovarian [14], colon [15], and liver [16] cancers. The CD133 levels are linked to the recurrence, metastasis, chemotherapy resistance, and radiation resistance [17]. A previous study on SCLC found that CD133^+^ cancer stem-like cells are highly tumorigenic and chemoresistant [18]. The glycoprotein showed resistance to chemotherapy in SCLC too; however, it may be an inadequate marker for the CSCs of SCLC [19]. Moreover, research on the impact of CD133 on the radiotherapy of SCLC is limited.

Herein, we have reported, for the first time, the expression pattern of the key Hippo pathway molecules in SCLC patients. In addition, we have assessed the modulation of radiation resistance by YAP1 and the impact of CD133 on radiotherapy for SCLC.

## 2. Materials and Methods

### 2.1. Clinical Samples and Immunohistochemical Staining

A total of 37 formalin-fixed, paraffin-embedded tissues were obtained from patients diagnosed with SCLC by bronchofiberscopy or biopsy between January 2012 and March 2015. All patients received care and follow-up in our hospital. Informed consent was obtained under the protocol approved by our hospital's ethics committee. The clinical characteristics are summarized in Table 1.

The endogenous peroxidase activity was blocked by soaking the deparaffinized specimens in 3.3% H_2_O_2_. The specimens were then incubated with primary antibodies (MST2, 1 : 200, ab87322, abcam; pMST2 (phosphor Thr180), 1 : 200, PA5-104616, Invitrogen; YAP1, 1 : 100, ab205270, abcam; pYAP1 (phosphor S127), 1 : 100,ab76252, abcam; CD133, 1 : 100, ab216323, Abcam) and the corresponding secondary antibodies [20]. Semiquantitative results were obtained by using the German semiquantitative scoring method, as previously described [21].

### 2.2. Cell Culture and Establishment of Stable Transfected Cells

Human H69 and H446 SCLC cell lines (ATCC) were used to prepare the cells overexpressing a constitutively activated form of YAP1, PEX2-FLAG-YAP1-5SA (GenePharma), which was transfected based on the provided directions. To establish cell lines expressing dominant negative YAP1, PEX2-FLAG-YAP1-5SA-^△^C (GenePharma) was transfected [21, 22]. PEX2 empty vector was utilized as a control. After selection in G418 for a month, stable transfections were established. All cells, including the sorted H69 CD133^+^ and H69 CD133^−^ cells, were incubated in RPMI1640 with 10% FBS (GIBCO, USA) and cultured in a humidified atmosphere of 5% CO_2_.

### 2.3. Cell Viability Assay

The cells were plated in 96-well plates (2 × 10^3^ cells/well) in triplicates for 6 h, after which they were irradiated (IR) at different doses. Following incubation with 10 *μ*L of Cell Counting Kit-8 solution (CCK-8; Dojindo, Japan) for 4 h, the absorbance at 450 nm was noted. The values were then expressed in percents by comparison with 0 Gy or 0 day, and the survival rates were accordingly calculated. The experiments were conducted in triplicates.

### 2.4. Colony Formation Assay

The cells were plated in 6-well culture plates at various densities and then subjected to different doses of IR exposure (100 cells-0 Gy; 200 cells-2 Gy; 500 cells-4 Gy; 1000 cells-6 Gy; 2000 cells-8 Gy; 5000 cells-10 Gy). The cells were subsequently cultured for 14 days. The survival fraction was calculated as the number of colonies divided by the number of cells seeded [23].

### 2.5. Western Blotting

The cells were lysed with radioimmunoprecipitation assay (RIPA) buffer (Sigma-Aldrich). After equilibration by the Pierce BCA assay, the lysates were resolved on 10% sodium dodecyl sulfate-polyacrylamide gel electrophoresis (SDS-PAGE), followed by electroblotting onto polyvinylidene difluoride (PVDF) membranes. The blots were then probed using primary anti-CD133 (1 : 500; Abcam), and the PVDF membranes were incubated with the appropriate horseradish peroxidase- (HRP-) linked secondary antibodies.

### 2.6. Flow Cytometry Analysis and CD133^+^ Cell Selection

The cells were treated with 6 Gy IR and detected using Annexin V/propidium iodide based on the furnished directions.

Next, PBS was used to wash the cells prior to resuspension at 1 × 10^5^/mL and stained using a polyethylene- (PE-) linked anti-human antibody (TMP4; Invitrogen). Finally, the cell suspension was subjected to flow cytometry for detecting the proportion of CD133^+^ cells, according to the manufacturer's instructions.

Anti-CD133 was used to stain the cells as mentioned above, and the top and bottom 7% of the stained cells were isolated. The purity of the sorted cells was evaluated using another CD133 antibody (EMK08; Invitrogen), as described earlier [18].

### 2.7. Statistical Analysis

A KaplanMeier approach was used to assess the survival, with death as the primary follow-up end point in the SCLC patients. Student's *t* or analysis of variance (ANOVA) tests were employed to calculate the *P* values for the data from the independent experiments. The quantitative data were expressed as mean ± standard deviation (SD). *P* < 0.05 was considered to be the significance threshold. All the statistical analyses were performed with the SPSS 19.0 software.

## 3. Results and Discussion

### 3.1. Expression of Key Hippo Pathway Proteins in the SCLC Patients

To analyze the importance of the Hippo pathway in SCLC, we examined the expression of MST2 and phosphorylated MST2 located upstream of the pathway in the specimens. We also studied the expression of YAP1 as well as its phosphorylated form located downstream and the core protein of the Hippo pathway in the SCLC specimens. The paraffin-embedded tissues were sectioned continuously to detect the expression of MST2, pMST2, YAP1, and pYAP1.

**Figure 1:**
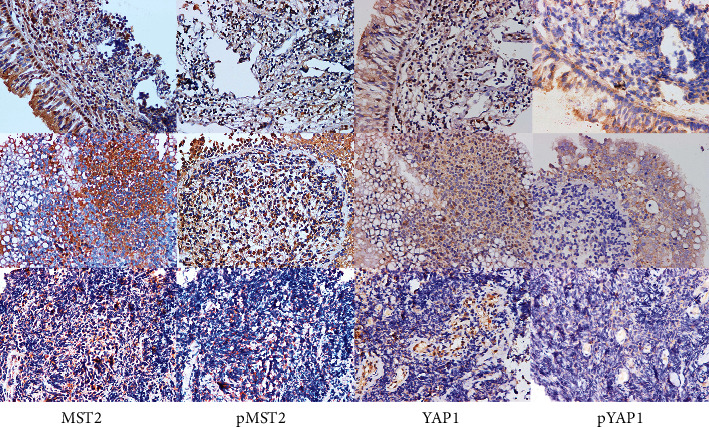
The expression of MST2, pMST2, YAP1, and pYAP1 in SCLC patients after the paraffin-embedded tissues were sectioned continuously (×400).

**Figure 2:**
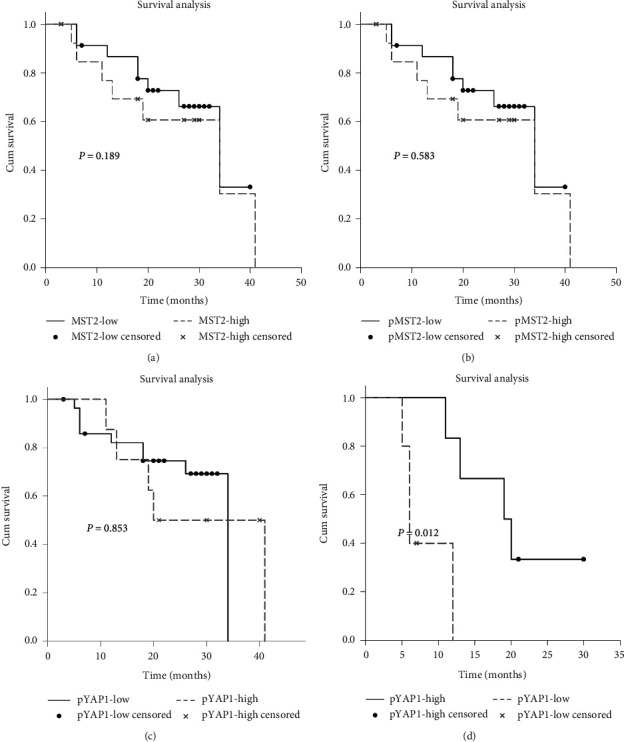
Prognostic analysis for MST2, pMST2, and pYAP1 performed on clinical samples. (a) Survival differences among MST2 high and low expression groups in SCLC patients assessed via a Kaplan–Meier approach (*P* = 0.189). Survival differences between (b) pMST2 high and low expressing groups (*P* = 0.583), (c) pYAP1 high and low expressing groups (*P* = 0.853), and (d) pYAP1 high and low expressing groups in SCLC patients with high YAP1 expression (*P* = 0.012).

As shown in Figure 1, MST2, pMST2, and pYAP1 were all detected mainly in the cytoplasm (the negative controls are shown in Figure S1). YAP1 was observed both in the nucleus and cytoplasm. The results of KaplanMeier analysis indicated that MST2, pMST2, and pYAP1 did not significantly correlate with SCLC patient's survival time (Figures 2(a)–2(c)). We had previously reported that patients with lower YAP1 expression survived longer than those with higher expression [21]. Considering that pYAP1 is the phosphorylated form of YAP1, we further investigated whether the expression of pYAP1 was correlated with the patient's survival time. The results implied that SCLC patients with low pYAP1 expression had a significantly poorer survival rate than those with high pYAP1 expression (Figure 2(d), *P* = 0.012).

As summarized in Table 1, the positive rates of MST2, pMST2, and pYAP1 staining were 40.54% (15/37), 37.84% (14/37), and 21.62% (8/37). Correlation analysis suggested that MST2, pMST2, and pYAP1 were not significantly associated with the age, gender, or the disease stage.

### 3.2. YAP1 Expression Is Associated with SCLC Radiation Resistance

To analyze the role of YAP1 in radiation resistance, we established the stable cell line H69-5SA overexpressing the constitutively active YAP1 and the stable cell line H446-5SA-△C with a dominant negative YAP1. On the other hand, H69-NC and H446-NC were used as controls [21, 22].

**Figure 3:**
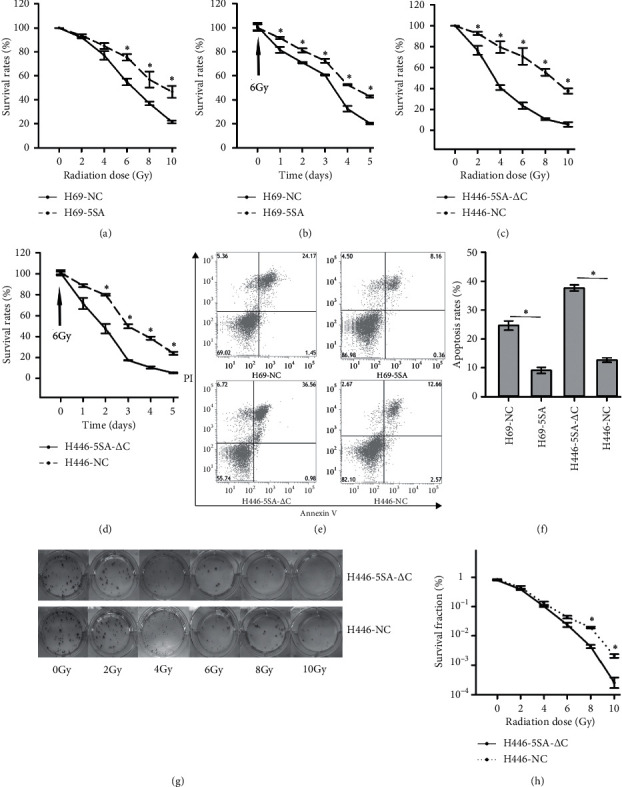
YAP1 may induce radiation resistance in SCLC. The survival rates of SCLC increased significantly (a) following different doses of IR when YAP1 was hyperactive and (b) at different time points after IR exposure of 6 Gy when YAP1 was hyperactive. The survival rates of SCLC decreased significantly (c) following different doses of IR when YAP1 was inhibited and (d) at different time points after IR exposure of 6 Gy when YAP1 was inhibited. (e) Representative pictures demonstrating the different survived rates after IR exposure of 6 Gy when YAP1 was hyperactive or inhibited. (f) Bar graphs showing the different survival rates after IR exposure of 6 Gy when YAP1 was hyperactive or inhibited. (g) Representative pictures demonstrating that a lower number of colonies survived and formed when YAP1 was inhibited following different doses of IR. (h) Line chart showing a lower number of colonies survived and formed when YAP1 was inhibited following different doses of IR.

We conducted the CCK-8 assay to detect the cell viability after IR exposure. The results demonstrated that the survival rates of H69-NC decreased significantly after different doses of IR (Figure 3(a)) or at different time points (Figure 3(b)) when compared with H69-5SA. Similar results were observed when YAP1 was inhibited (Figures 3(c) and 3(d)). We then conducted flow cytometric analysis to evaluate the apoptotic rates after IR exposure of 6 Gy. The rates in H69-NC and H446-5SA-△C were significantly higher than in H69-5SA and H446-NC (Figures 3(e) and 3(f)). H69-5SA and H69-NC were used as the suspension cells. Hence, H446-5SA-△C and H446-NC were used for the colony-forming assay, which suggested that the survival fraction decreased significantly when YAP1 was inhibited after IR exposure in different doses (Figures 3(g) and 3(h)).

### 3.3. CD133 May Be Associated with the Radiation Resistance of YAP1

**Figure 4:**
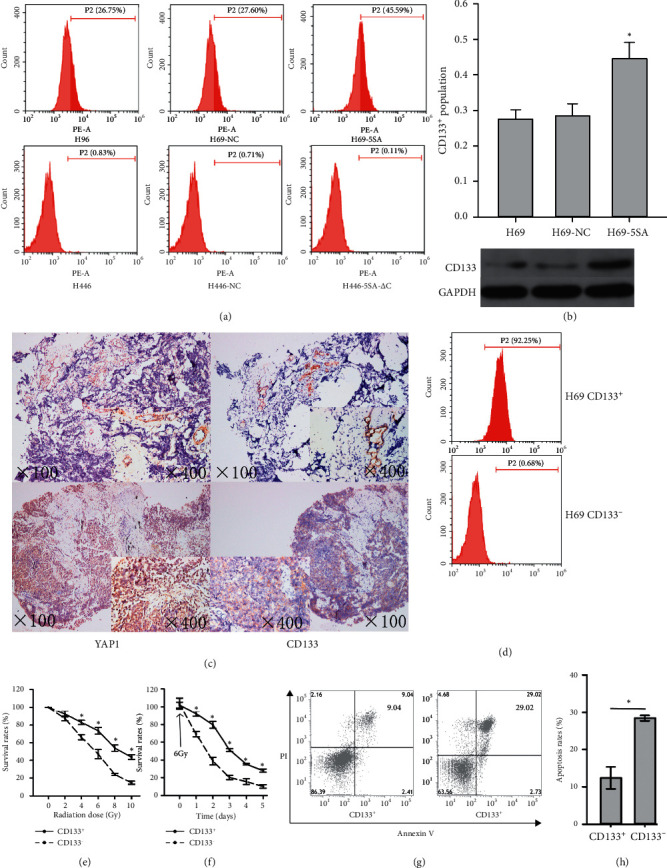
CD133 may be controlled by the expression of YAP1 and CD133 may be associated with the radiation resistance of SCLC. (a) The percent of CD133^+^ changed when YAP1 was hyperactive or inhibited in SCLC. (b) The percent of CD133^+^ increased significantly when YAP1 was hyperactive. (c) The expression of YAP1 and CD133 in SCLC patients after the paraffin-embedded tissues were sectioned continuously. (d) The percent of CD133^+^ after CD133^+^ cells selection in H69. The survival rate of CD133^+^ increased significantly (e) following different doses of IR as compared to that of CD133^−^ and (f) at different time points after IR exposure of 6 Gy as compared to that of CD133^−^. (g) Representative pictures demonstrating the different survival rates between CD133^+^ and CD133^−^cells. (h) Bar graphs showing the different survival rates between CD133^+^ and CD133^−^ cells.

Flow cytometric analysis was conducted to evaluate the proportion of CD133^+^ in SCLC cells. Our findings implied that the proportion of CD133^+^ increased significantly in H69-5SA relative to H69-NC and H69 (Figures 4(a) and 4(b)). Western blotting also yielded similar results (Figure 4(b)) (the full western blot is shown in Figure S2). The proportion of CD133^+^ was relatively lower in other cells than that in H69 (Figure 4(a)). The tissues were sectioned continuously to detect YAP1 and CD133 expression in the SCLC patients (Figure 4(c)) (the negative control of YAP1 and CD133 are shown in Figure S1). As represented in Table 2, the expression of CD133 and YAP1 exhibited significant correlation (*P* = 0.008). After sorting and isolation using CD133 microbeads separation system, the proportion of H69 CD133^+^ and H69 CD133^−^ was evaluated by flow cytometric analysis (Figure 4(d)). Later, we checked whether CD133 was associated with the radiation resistance of SCLC. The results of cell viability assay (Figures 4(e) and 4(f)) and flow cytometric analysis (Figures 4(g) and 4(h)) revealed that the subpopulation of CD133^+^ cells displayed higher radiation resistance than the CD133^−^ cells.

## 4. Conclusions

Although systemic therapies including immunotherapy have begun to show promising outcomes in the past few years, the outcomes of SCLC patients remain to be substantially impacted [24]. Chemotherapy and radiation therapy are still the main stay approaches regardless of the stage of LD or ED [25]. Therefore, it is vital to bridge our knowledge gaps in understanding the underlying mechanisms of chemotherapy and radiation resistance.

A genome-wide single nucleotide polymorphism (SNP) scan detected that the SNP within the YAP1 promoter is associated with SCLC survival [26]. Our previous study indicated that the phosphorylation of MST2 and YAP1 may modulate the multidrug resistance of SCLC [20]. One large-scale screening dataset asserted that a YAP/TAZ subgroup in the SCLC cell lines may be more susceptible to chemotherapy or targeted therapies [27]. Two SCLC subgroups were spotted based on reciprocal INSM1 and YAP1 expression in the SCLC cell lines [28, 29]. The low neuroendocrine subtypes of SCLC played a more significant role in activating the Hippo pathway when compared with the high neuroendocrine subtype [30]. We previously determined that YAP1 expression is correlated with the patient's disease stage and survival. We first reported YAP1 to be capable of inducing multidrug resistance in SCLC both under in vitro and in vivo conditions [21]. However, the expression of other key proteins in the Hippo pathway, such as MST2, pMST2, and pYAP1 remain to be researched. In this study, we documented the expression of MST2, pMST2, and pYAP1 in the SCLC patients and found that the pYAP1 expression may be associated with the survival time of patients with high levels of YAP1. This observation suggests that the expression of pYAP1 may be valuable in the treatment of the “YAP/TAZ subgroup” in the future. In this study, we proved that YAP1 plays a role in cell viability and influences the apoptosis and proliferation ability after IR exposure, suggesting that the protein may induce radiation resistance in SCLC.

CD133 contributes to impaired patient survival as well as increased tumor progression and recurrence in many cancers. Its expression is regulated by factors such as epigenetics, signaling, and the tumor microenvironment [17]. However, not much is known about CD133 and the Hippo pathway. In this study, we unearthed that the proportion of CD133 in the SCLC cells is controlled by the expression of YAP1 based on the results of western blotting and flow cytometry. The expression of CD133 and YAP1 in SCLC patients demonstrated significant correlation as well. We then sorted and isolated the CD133^+^ and CD133^−^ cells in H69 and found that CD133 may be linked with the radiation resistance of SCLC.

In this research, we have reported for the first time the expression of key Hippo pathway proteins in the SCLC patients. The expression of pYAP1 appears to be significant to the “YAP/TAZ subgroup”. Moreover, CD133 may be controlled by the expression of YAP1 in the Hippo pathway, and it may be associated with the radiation resistance of SCLC.

## Figures and Tables

**Table 1:** The expression of MST2, pMST2, pYAP1, and their relationships with the clinicopathological characteristics in SCLC patients.

Variables	Total number *n* = 37	MST2 expression		*P*	pMST2 expression		*P*	pYAP1 expression		*P*
		Low	High		Low	High		Low	High	
		22	15		23	14		29	8	
Age				0.385			0.420			0.931
≤56	18	12	6		10	8		14	4	
>56	19	10	9		13	6		15	4	
Gender				0.683			0.595			0.862
Male	33	20	13		21	12		26	7	
Female	4	2	2		2	2		3	1	
Stage				0.476			0.869			0.098
LD	27	17	10		17	10		23	4	
ED	10	5	2		6	4		6	4	

**Table 2:** The expression of YAP1 and their relationships with CD133 in SCLC patients.

Variables	Total number	YAP1 expression		Fisher's exact test
	*n* = 37	Low	High	*P* value
		*n* = 26	*n* = 11	
CD133 expression				
Low	25	21	4	0.008
High	12	5	7	

## Data Availability

The data used to support this study are available from the corresponding author upon request.

## Supplementary Materials

Supplementary Materials Figure S1: the negative control of the expression of MST2, pMST2, YAP1, pYAP1, and CD133 (×400). Figure S2: the representative image of full western blot of Figure 4(b).
