# Supplementary material for: Evaluation of Hippo Pathway and CD133 in Radiation Resistance in Small-Cell Lung Cancer
Source: J Oncol. 2021 Jan 13;2021:8842554. doi: 10.1155/2021/8842554 (PMC7817273; doi:10.1155/2021/8842554)
Supplement: Supplementary Materials — Figure S1: the negative control of the expression of MST2, pMST2, YAP1, pYAP1, and CD133 (×400). Figure S2: the representative image of full western blot of Figure 4(b). [file 8842554.f1.zip › 8842554.f1/Figure S2.doc]

Figure S2. The representative image of full western blot of Figure 4B.
